# Viscum Album — A Possible Treatment of Cancer?

**Published:** 1973-01

**Authors:** M. R. Evans, A. W. Preece

**Affiliations:** University of Bristol; University of Bristol


### *Viscum album* — A possible treatment for Cancer?

**M. R. Evans and A. W. Preece**
**University of Bristol**

### Introduction

There are a number of reports in the literature—only a few of which are in English language—suggesting a cancerostatic action for a Mistletoe extract. We have reviewed the literature on the subject and carried out limited experimental confirmation of one of the findings, using Iscador Q—a proprietary preparation of *Viscum album*. We hope that this may prompt a more thorough investigation and further work on this subject.

The use of mistletoe was first suggested specifically for the treatment of cancer by Steiner (1926). From this stemmed the preparation 'Iscador' which is extracted from the juice of the whole plant and what follows includes work on this as well as on specific fractions from freshly prepared juice.

### Vester's Work

An analysis of freshly prepared juice of *Viscum album* showed a high content of free amino acids with an unexpected predominance of basic amino acids and an absence of some acidic amino acids (Vester, 1965). The distribution pattern was suggested to be complementary to those found in fish which have a high rate of spontaneous tumour formation. *Viscum album* was found to contain several 'cancerostatic' components, the most active one occurring in the native protein fraction, while another was found in the protein free fraction. Tumour inhibition was demonstrated in mice carrying subcutaneously inoculated sarcoma 180 as well as in cell cultures of sarcoma 180, HeLa and Chang liver cells (Vester and Mai, 1960; Selawry et al., 1961).

The purification procedures for the active fraction were fully described by Vester. The active components were found to be histone-like, basic proteins, molecular weight approximately 60,000 (Vester et al., 1968a).

The *in vivo* and *in vitro* effect are thought to be different, *in vivo* effect being abolished on heat denaturation whereas the *in vitro* effect is not. In testing a variety of preparations of *Viscum album* on mice bearing sarcoma 180 tumours the best results were obtained from preparations of the whole plant as opposed to a single fraction, the treated tumour weight/control tumour weight being 22% and 28% respectively at the maximum tolerated doses for five days. Vester describes a long series of physical and chemical separation procedures which yielded a large number of different protein fractions. These were tested against Crocker sarcoma 180 in mice and cell cultures. The most effective fraction produced a 50% reduction in tumour size (compared with controls) at a dose of only 0.5 μg/Kg. An average molecular weight of 60,000 means an active dose of $8.3 \times 10^{-15}$ mols/g. (He also quotes a figure of $8.5 \times 10^{-11}$ mols/g for the commercial preparation Iscador.)

In testing the various fractions for the effects in producing tumour inhibition *in vivo*, toxic effects *in vivo*, inhibition of cell cultures and of both HeLa cells and amnion cells, there is little correlation between the presence or absence of the various effects from fraction to fraction, suggesting a variety of different proteins with different properties. Some of the protein components were found to be strongly antigenic, since less than 10 μg induced rapid antibody formation in rabbits. Vester suggests that this may be less important in human patients with tumours who may have a reduced immunological response.

The denaturing effects of pH and heat were examined and it was shown that the *in vivo* tumour inhibiting effect was easily destroyed, the general toxic effect being increased in some cases. Denaturing has far less effect on the *in vivo* activity. The proteins responsible for the tumour inhibiting effect were thought to be unusually labile (Vester et al., 1968b). The effect of one of the active fractions of *Viscum album* (No. 16) on an *in vitro* preparation of Yoshida ascites cells was examined, monitoring R.N.A. (ribonucleic acid) synthesis by the uptake of labelled uridine tri-phosphate. At an optimal dose it was reduced to 30%. Mild denaturation (2 hours at 50°C) of the fraction before testing enabled a smaller dose to produce the same effect, and at the optimal dose uptake was reduced to 25%. This was thought to be as a result of the loss of specificity for only certain parts of the D.N.A. (desoxyribonucleic acid) although it is difficult to see how the one molecule per chromosome could produce such a large reduction in D.N.A. synthesis. Inhibition of R.N.A. and D.N.A. production was demonstrated in HeLa cells and fibroblasts but the mode of response gave little clarification as to the underlying mechanism (Vester et al., 1968c; Klamerth et al., 1968).

### Theories as to method of action

1. It is felt unlikely that the action of *Viscum* extract is primarily that of an antimetabolite or a mitotic poison because of the lack of correlation between the tumour inhibiting effects, the toxic effects and the inhibition of different cell cultures by the various protein fractions. In addition, there was a lack of the usual side-effects associated with cytotoxic therapy.

2. The stimulation of the immunological response of the organisms has been suggested. This is supported by the rise in antibody titre in rabbits, by the production clinically of a temporary leucocytosis and mild pyrexia and stimulation of the cell mediated response suggested by thymic enlargement (see under toxicity). This may not be the whole explanation.

3. Vester postulates a direct effect on aberrant cell controlling mechanisms, using the model of Jacob and Monod (1961). In this model, nucleoproteins are thought to repress certain lengths of D.N.A. thereby maintaining differentiation, a characteristic of cells not behaving cancerously. In a hybrid fish, Poeciliidae, known for its high rate of spontaneous tumour development, it is thought that nucleoproteins are unusually acidic, making their attachment to the D.N.A. less stable. This instability makes it difficult to maintain differentiation and may explain the high rate of spontaneous tumour formation. It is at the level of these processes that the mistletoe protein may act. This is supported by the following evidence:—
   (a) The structural similarity of the active fractions to histones.
   (b) Their basic reaction, allowing direct and firm attachment of the molecule to the nucleic acid.
   (c) The small number of molecules required per cell, calculated to be less than 38.
   (d) D.N.A. was the only one of a series of metabolites found to inhibit the cancerostatic action of these proteins.
   (e) The sensitivity to denaturation. The specific cancer inhibiting property becomes a general cytotoxic property. This is as one might expect in a protein claimed to have such an an extreme specificity of action.

**Cancer Chemotherapy National Service Centre report**

The C.C.N.S.C. report finding preliminary activity in K.B. cell culture (human epidermoid carcinoma of the nasopharynx, in water) using crude extracts of *Viscum album*. An E.D. 50 of 0.74 $\mu$g/ml was established. When tested on Sarcoma 180 in mice at optimal doses, T/C ratios of 56%, 60%, 51% and 54% were achieved in various trials. Further work was not done as the extract failed to achieve a T/C ratio of 42% or less (C.C.N.S.C. 1962).

**Authors' results**

We tested the claim (Vester 1968) that the growth of Crocker sarcoma 180 is inhibited by doses of Iscador 8.5 x $10^{-11}$ mol/g body weight, calculated on the assumption of an active component of molecular weight 60,000, to the effect that at the end of a ten-day test period the Iscador treated mice carried tumours of 50% of the weight of tumours in control animals. Our results confirmed that doses of Iscador of the order of magnitude quoted by Vester do indeed have a tumour inhibiting effect ($p<0.005$), though our results suggest that the degree of inhibition may be somewhat less than 50%. The Crocker 180 Sarcoma, because of its lack of specific antigenicity, is not an ideal model with which to attempt to mimic the behaviour of spontaneous human tumours. A far better model may be the primary carcinogen-induced tumours but as far as we are aware, no work relating to Iscador has yet been carried out on tumours of this type.

**Toxicology**

Using an active fraction of *Viscum album* the L.D.50 was determined in mice and found to be about ten times greater than a therapeutic dose for this fraction (Vester, 1968; author's unpublished data). Post mortem examination of these animals revealed haemorrhagic effects in many organs. These effects disappeared at levels between the L.D.50 and the therapeutic level. At levels around the "therapeutic range" 200 necropsies revealed splenic and thymic enlargement, the thymus showing hyperplasia microscopically. Similar effects were found with the proprietary preparation Iscador. Stimulation of the thymus with its relation to the cell-mediated immunity may therefore also be of therapeutic importance. This further complicates interpretation of its mode of action. Clinically Iscador Nienhaus et al 1970 is found to cause a mild pyrexia and mild leucocytosis. This is used to monitor the dose given. An increase in thermic, inflammatory reactions has been noted in lymph nodes and the region around certain tumours. It lacks the toxic effects of traditional cytotoxic drugs in all aspects. In over 1,000 treated patients, neither Gunczler and Salzer (1969) nor Fellmer (1967) report any anaphylactoid reactions or other serious side effects.

*See Appendix for full results.

**Clinical Experience**

1. At the Munich University Hospital, Fellmer (1967) treated 81 patients with carcinoma of the *cervix*, and compared the results with a large control group of 709 patients. The five year survival rate of the control group when corrected for those who died in the first few weeks was 69% compared with the five year survival rate of 83% in the treated group. Both groups were initially treated with radiotherapy and the experimental group was given Iscador as a follow-up treatment.

Only stages 1, II and III were included in either group and the relative distribution by stages was:—

| | I | II | III |
|---|---|---|---|
| Control | 1 | 3.3 | 2.8 |
| Iscador treated | 1 | 4.5 | 2.7 |

Although the two groups have a similar stage distribution, there is no account of a thorough randomisation procedure at the outset.

2. At the lainz Hospital Vienna, Gunczler and Salzer (1969) have used Iscador in the post operative treatment of carcinoma of the breast, stomach, rectum and colon.

*Breast*—257 treated patients are compared with a control group of 187. At 2 years there was a statistically significant improvement of survival by about 8% but none at 5 years. The control groups were those patients operated on in a previous period when radical mastectomies were performed on all patients, whereas many of the treated group had either partial resections or simple mastectomies. It could be argued that the difference at two years reflected only the severity of surgery.

*Stomach*—67 treated patients are compared to 101 control patients. It is stated that the treated group contained more patients with poor prognoses. The five year survival rate is over 10% higher in the treated group but not statistically significant. It is interesting to note that, if only those with lymph node involvement are compared from each group the difference in five year survival between the groups is over 20% and is statistically significant.

*Rectum*—This phenomenon is repeated in the results of treating carcinoma of the rectum. There is only a very slight difference between the total groups. Of those with lymph node involvement five out of fourteen treated patients survived five years, but none of the seven untreated patients (obviously very small numbers).

*Colon*—In the post operative treatment of carcinoma of the colon (excluding rectum) 47 treated patients are compared with 91 control patients. The treated group shows a 74% five year survival compared with a 49% survival in the controls. The control and experimental groups are claimed to have a very similar staging distribution. The Iscador treated patients show a statistically significant better survival rate throughout the five year follow-up.

### Conclusion

The evidence suggests that Iscador may well have a useful therapeutic effect on tumours. This is supported by evidence of some activity in animal experiments. It would also appear that this is not a consistent, radical or curative treatment and the effects on animal tumours is small compared with any cytotoxic agents used at optimal doses.

Clinically it is claimed, and this is to some extent supported by animal experiments, that Iscador does not possess the side-effects inherent in the usual cytotoxic agents. In fact, the reverse occurs. There is a stimulation of the humoral and cell-mediated immunological system, rather than the suppression occurring with cytotoxic therapy.

It may have a possible specific effect on the genetic material of tumour cells—but the evidence and mechanism for this is as yet far from clear.

In spite of its relatively weak anti-tumour effects it may be a preparation that could usefully be used in the long term treatment of cancer patients alongside surgery and radiotherapy and not be reserved for the more limited role of the majority of current chemotherapeutic agents due to their relative non-specificity and toxicity.

### Bibliography

C.C.N.S.C. Screening Data Summaries on Preparations B.99720 (1962) B.606001 (1966) B.656001 and B.666001 (1966) (Personal communication).

FELLMER, K. E. (1967) Nachbehandlung bestrahlter Genitalkarzinome mit dem Viscum-album-Praparat Iscador(R) zur Rezidivprophylaxe. Medizinische Klinik, **62**, 305.

GUNCZLER, M., G. SALZER (1969) Iscadortherapie in der Nachbehandlung operierter Carcinome. Osterreichische Arzterzeitung, **20**, 2290.

JACOB, F. and MONOD, J. (1961), Genetic regulating mechanism in the synthesis of protein. J. molec. Biol, **3**, 318.

KLAMERTH, O., F. VESTER, G. KELLNER (1968) Inhibitory effects of a protein complex from Viscum album on fibroblasts and HeLa cells. Hoppe-Seyler's Z. Physiol. Chem., **349**, 863.

NIENHAUS, J. M., M. STOLL, F. VESTER (1970) Thymus stimulation and cancer prophylaxis by Viscum proteins. Experientia, **26**, 523.

SELAWRY, O. S., F. VESTER, W. MAI, M. R. SCHWARTZ (1961) Zur Kenntnis der Inhaltsstoffe von Viscum album II, Tumorhemmende Inhaltsstoffe. Hoppe-Seyler's Z. Physiol. Chem., **324**, 262.

STEINER, R. (1924). Geisteswissenschaft und Medezin. Rudolf Steiner Press, London. p. 70.

VESTER, F. (1965) Amino Acid Pattern and Malignant Growth. Biochim. Biophys. Acta., **104**, 98.

VESTER, F., W. MAI (1960) Zur Kenntnis der Inhaltsstoffe von Viscum album I, Frei Aminosauren. Hoppe-Seyler's Z. Physiol. Chem., **322**, 273.

VESTER, F., A. STEEL, M. STOLL, M. MULLER (1968a) Zur Kenntnis der Inhaltsstoffe von Viscum album III, isolierung und Reinigung cancerostatischer Proteinfraktionen. Hoppe-Seyler's Z. Physiol. Chem., **349**, 125.

VESTER, F., L. BOHNE, M. EL-FOULY (1968b) Zur Kenntnis der Inhaltsstoffe von Viscum album IV, Biologisches Verhalten einzelner Proteinfraktionen. Hoppe-Seyler's Z. Physiol. Chem., **349**, 495.

VESTER, F., A. SCHWEIGER, A. SEEL, M. STOLL 1968c) Die Hemmwirkung basischer Proteine aus Viscum album auf die R.N.A.-Synthese in Yoshida-Ascites. Hoppe-Seyler's Z. Physiol. Chem., **349**, 865.

Many of these papers are in German but a limited number of translations of some of them are available on request from the authors of this article.

We wish to thank Dr. R. Leroi of the Verein fur Krebsforschurg, Arlesheim, Switzerland for providing samples of Iscador.

### APPENDIX

### Inhibition of growth of Crocker 180 Sarcoma by Viscum Album (Iscador)

### Introduction

It has been reported (Vester, 1968) that the growth of the Crocker 180 Sarcoma is inhibited by doses of Iscador 8.5 x $10^{-11}$ mol/g body weight, calculated on the basis of an active component of molecular weight 60,000. At the end of a 10 day test period, Iscador treated mice carried tumours of 50% of the weight of tumours on control animals. It has been stated that the effective dose is related to the toxic dose of the compound.

### Method

In this trial the optimal dose level was determined on a small group of animals, and then repeated for a given dose level on further batches of mice. Figures for the L.D. 50 of .15 ml of 4% have been quoted by Neinhaus et al. (1970). The L.D.50 for the strain of mice used here was determined independently because of possible strain-specific differences in sensitivity.

The tumour used was a high passage Crocker 180 Sarcoma implanted in random bred male Balb/c mice. Animals were selected from a narrow weight range rather than age to ensure uniformity of dose level. A fragment of tumour (approx. 1 cmm) was subcutaneously transplanted in the right flank endeavouring to obtain uniformity of size of implant. After random selection into control of experimental groups, experimental animals were given daily I.P. doses of 0.15 ml of Iscador of different concentration.

On day 9 both control and experimental groups were killed and tumours dissected out for weighing.

The average weights of tumours from control and experimental groups were compared to determine the T/c ratio.

**Results**

**1—Finding the 'optimal' dose level**

| | Dose of Iscador | No. expt. mice | No. control mice | T./C. rates |
|---|---|---|---|---|
| a. | .15 ml of 0.4% | 6 | 6 | 86% |
| b. | .15 ml of 0.2% | 6 | | 50.7% |
| c. | .15 ml of 0.1% | 6 | 6 | >100% |
| d. | .15 ml of 0.05% | 6 | | 93% |

Due to a relatively short supply of mice the optimal dose level was not established any more accurately than being .15 ml of 0.2%.

**II—Detailed results using doses of .15 ml of 0.2% Iscador**

1. (Ib amplified)
   Mice: 6 test 6 controls (weights 25-35 g)
   Average weight of control tumours=.369 g
   Average weight of treated tumours=.187 g
   T/C ratio = 50.7%
   S.D. = .149 $(x_1—x_2)$ = .182; t=2.12
   p = <0.1

2. Mice: 9 test 8 controls (weights 30-33 g)
   Average weight of control tumours=.159 g
   Average weight of treated tumours=.086 g
   T/C ratio = 54%
   S.D. = .071 $(x_1—x_2)$ = .073; t=1.72
   p = 0.1

3. Mice: 12 test 12 controls (weight 28-32 g)
   Average weight of control tumours=.294 g
   Average weight of treated tumours=.231 g
   T/C ratio = 78.5%
   S.D. = .214 $(x_1—x_2)$ = .063; t=72
   p = <0.5.

**Results over all:**

Average T/C = 64% ± 15%

Overall probability of achieving these results b[illegible] chance p = 0.1 x 0.1 x 0.5 = 0.005

i.e. less than 1 in 200

**Discussion:**

The optimal dose determined experimentally is abo[illegible] 0.15 ml of 0.2% equivalent to .11 mg (dry weigh[illegible] compared to Vester's .153 mg for a 30 g mous[illegible] Our dose though slightly less, is clearly of the sam[illegible] order of magnitude.

The overall probability is achieved by multiplyi[illegible] individual probabilities. The results show a statistica[illegible] significant inhibition of tumour growth, though t[illegible] degree of inhibition cannot be accurately assessed d[illegible] to the variability, and the relatively small degree [illegible] inhibition, which on the basis of the rate of growth o[illegible] represents about 1 day delay. It appears to achie[illegible] T/C ratio between 50%-75%.

**Conclusion**

These experiments confirm that doses of Iscad[illegible] of the order of magnitude quoted by Vester do inde[illegible] have a tumour-inhibiting effect though they sugg[illegible] that the degree of inhibition may be somewhat le[illegible] than 50%.

